# Mortality and incidence of second cancers following treatment for testicular cancer

**DOI:** 10.1038/sj.bjc.6603589

**Published:** 2007-01-30

**Authors:** D Robinson, H Møller, A Horwich

**Affiliations:** 1 King's College London, Thames Cancer Registry, London SE1 3QD, UK; 2 Academic Unit of Radiotherapy and Oncology, Institute of Cancer Research, Downs Road, Sutton, Surrey SM2 5PT, UK

**Keywords:** testicular cancer, seminoma, nonseminoma, survival, multiple primaries

## Abstract

We studied 5555 seminoma patients and 3733 patients with nonseminomatous testicular cancers diagnosed in Southeast England between 1960 and 2004. For both groups survival improved over time: 10-year relative survival increased from 78% in 1960–1969 to 99% in 1990–2004 for seminomas, and from 55 to 95% for nonseminomas. In the early period mortality was still significantly increased more than 15 years after diagnosis in both groups, whereas in more recent periods the excess deaths mainly occurred in the first 5 years after diagnosis. For seminomas, there was a significant excess of cancers of the colon (standardised incidence ratio (SIR) 2.36; 95% confidence interval (CI) 1.13–4.35), soft tissue (SIR 13.64; CI 1.65–49.28) and bladder (SIR 4.28; CI 2.28–7.31) in the long term (20+ years after diagnosis), of pancreatic cancer in both the medium (10–19 years) (SIR 2.91; CI 1.26–5.73) and long term (SIR 5.48; CI 2.37–10.80), of leukaemia in both the short (0–9 years) (SIR 3.01; CI 1.44–5.54) and long term (SIR 4.48; CI 1.64–9.75), and of testis cancer in both the short (SIR 6.69; CI 4.28–9.95) and medium term (SIR 3.96; CI 1.08–10.14). For nonseminomas, significant excesses were found in the long term for cancers of the stomach (SIR 5.13; CI 1.40–13.13), rectum (SIR 4.49; CI 1.22–11.51) and pancreas (SIR 10.17: CI 3.73–22.13), and for testis cancer in the medium term (SIR 5.94; CI 2.18–12.93). Leukaemia was significantly increased in the short term (SIR 6.78; CI 2.93–13.36). The better survival observed is largely attributable to improved treatment, and the trend in reducing the toxicity of therapy should continue to reduce future health risks in testicular cancer survivors.

Testicular germ cell cancer occurs predominantly in young adulthood and is highly curable (Coleman *et al*, 2003). This high cure rate heightens concern over the risk of late treatment effects including second primary cancers (Fossa, 2004; Zagars *et al*, 2004), of which an increased incidence has been found following both radiotherapy and chemotherapy (Travis *et al*, 2005). This risk may depend upon specific details of treatment such as the chemotherapeutic drugs used or choice of radiation dose and field; a previous study from the Thames Cancer Registry (TCR) based on 859 patients treated with radiotherapy for seminoma between 1961 and 1985 did not find an increase in mortality risks from other causes (Horwich and Bell, 1994). In this report we extend the previous TCR study, assessing the overall mortality and incidence of second primary cancers in 9892 patients with first testis cancer diagnosis between 1960 and 2004.

## PATIENTS AND METHODS

Details of all testicular cancers (ICD-10 code C62) diagnosed between 1960 and 2004 were extracted from the TCR database. Of the 9941 testis cancers identified, nine were the contralateral cancers of bilateral pairs diagnosed on the same day, and 40 were second primaries diagnosed subsequent to an earlier testicular cancer. We thus had an index cohort of 9892 first testis cancer cases. These were subsequently categorised on the basis of their morphology as seminomas, nonseminomas, or of other/unspecified type; the latter group excluded lymphomas.

The TCR is a population-based registry covering London and a large part of Southeast England, with a resident population at the time of this study of approximately 14 million. Patients registered at TCR represent a cohort of individuals followed up from cancer diagnosis to death. Those diagnosed before 1 January 1971 were followed up actively to obtain death information until 31 December 1982, and were censored at this date. Patients diagnosed from 1 January 1971 onwards were followed up passively through the National Health Service (NHS) Central Register, which provides notification of all deaths routinely to the registry. These patients were censored at 31 December 2004 (the date up to which we could assume death information to be essentially complete).

Person-years at risk were calculated from the date of diagnosis of testicular cancer to the date of the event of interest (either death or first diagnosis of cancer at a specific site) or to the date of exit from the study (date of death or loss to follow-up, or censoring date as defined above, whichever was the earlier).

Relative survival was estimated by the method of Estève *et al* (1990), comparing observed mortality with expected mortality derived from life tables for Southeast England (Coleman *et al*, 1999). In addition, observed and expected numbers of deaths (derived from the same life tables) in each of three periods of follow-up (0–4, 5–14, and 15+ years after diagnosis) were compared to give standardised mortality ratios (SMRs). For the incidence of subsequent cancers, male age/calendar period specific cancer incidence rates for the TCR region were applied to the cohort to calculate the expected number of subsequent tumours at each site. From the observed and expected numbers standardised incidence ratios (SIRs) were derived. Exact 95% confidence intervals (CIs) were calculated assuming a Poisson distribution for the observed numbers. All statistical calculations were performed using the statistics package Stata (Statacorp, 2003).

## RESULTS

**Figure 1:**
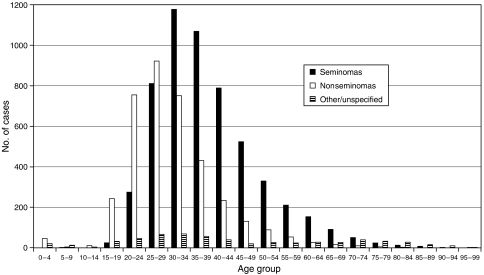
Age distributions by testis cancer type.

Of the 9892 first testicular cancers, 5555 (56.2%) were seminomas, 3733 (37.7%) nonseminomas, and 604 (6.1%) other/unspecified. Mean ages at diagnosis were 39.3 (s.d. 11.4) years for seminomas, 31.0 (s.d. 10.4) years for nonseminomas, and 45.0 (s.d. 23.3) years for the other/unspecified group. Figure 1 shows the age distributions in these three groups. Seminoma cases were in general older than those with nonseminomas. The relatively small group of ‘other/unspecified’ cases have been excluded from subsequent analyses. A total of 1590 deaths (820 in seminoma cases and 770 in nonseminomas) occurred during a total of 104 622 person–years of follow-up. Only 51 cases were known to be lost to follow-up.

**Figure 2:**
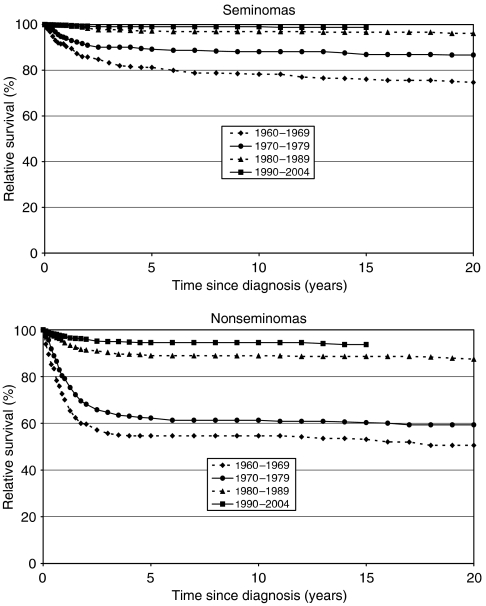
Relative survival by period of diagnosis.

Figure 2 shows relative survival in seminomas and nonseminomas for up to 20 years after diagnosis, stratified by period of diagnosis. In all periods, survival was better in seminomas. For both groups survival improved over time, particularly for nonseminomas. Ten-year relative survival estimates increased from 78% in 1960–1969 to 99% in 1990–2004 for seminomas, and from 55 to 95% for nonseminomas over the same period.

Table 1 shows SMRs and 95% CIs during the three periods of follow-up, again stratified by period of diagnosis. In keeping with the relative survival figures, the SMRs for both types of cancer decreased over time. In the very early period (1960–1969) a significantly increased mortality persisted more than 15 years after diagnosis in both seminomas and nonseminomas. However, in more recent periods the majority of excess deaths were in the first 5 years after diagnosis. For cases diagnosed between 1990 and 2004, the SMR in this first 5-year period was 1.69 (95% CI 1.36–2.08) for seminomas and 8.50 (95% CI 7.03–10.19) for nonseminomas.

The results relating to subsequent cancers are shown in Tables 2 and 3, with length of follow-up stratified into 0–9, 10–19, and 20+ years following diagnosis. A specific cancer site was included in these tables if the total number of subsequent cancers at this site exceeded 10. Sites satisfying this criterion were stomach (C16), colon (C18–C19), rectum (C20–C21), pancreas (C25), lung (C33–C34), prostate (C61), testis (C62), kidney (C64), bladder (C67), non-Hodgkin's lymphoma (C82–C85), and leukaemia (C91–C95). Also included in the table were malignant neoplasms of ‘other connective and soft tissue’ (C49), as a means of identifying soft tissue sarcomas. All remaining sites (with the exception of nonmelanoma skin cancers (C44), which are poorly recorded) were grouped into an ‘other’ category.

A total of 409 cancers occurred subsequent to testis cancer (excluding nonmelanoma skin cancers and testis cancers), 296 after seminomas and 113 after nonseminomas. For seminomas, there was a significant excess of cancers of the colon, soft tissue and bladder and of ‘other’ cancers in the long term (20+ years after diagnosis). In addition, there was an excess of pancreatic cancer in both the medium (10–19 years) and long term, of leukaemia in both the short (0–9 years) and long term, and of testis cancer in both the short and medium term. Lung and ‘other’ cancers were significantly reduced in the short term. For nonseminomas, significant excesses were found in the long term for cancers of the stomach, rectum and pancreas, and for testis cancer in the medium term; leukaemia was significantly raised in the short term.

Sites included in the above-mentioned ‘other’ group which showed significant excesses were: anus (C21) (SIR 7.98; CI 1.12–56.64), bone and articular cartilage other than of limbs (C41) (SIR 25.96; CI 3.66–184.28), renal pelvis (C65) (SIR 17.34; CI 2.44–123.09), and Hodgkin's disease (C81) (SIR 12.99; CI 3.25–51.93) in seminoma patients and C21 (SIR 15.44; CI 2.17–109.58), heart, mediastinum and pleura (C38) (SIR 40.84; CI 5.75–289.94), C41 (SIR 47.57; CI 6.70–337.69) and skin melanoma (C43) (SIR 4.30; CI 1.08–17.19) in nonseminoma cases. These excesses were all seen more than 20 years after diagnosis.

## DISCUSSION

The data in this report illustrate an overall improvement in survival during the study period, which is likely to be mainly because of improvements in treatment, especially the development of effective combination chemotherapy during the 1970s (Einhorn and Donohue, 1977; Peckham *et al*, 1983). Improved survival in the first 5 years after diagnosis was evident even in comparisons of those diagnosed between 1980 and 1989 with those diagnosed between 1990 and 2004 (Table 1). This may be owing to the trend to presentation of germ cell tumours at an earlier stage (Sonneveld *et al*, 1999; Powles *et al*, 2005). For seminomas the SMR improved from 2.59 (95% CI 2.02–3.28) to 1.69 (CI 1.36–2.08) and for nonseminomas from 15.09 (CI 12.37–18.24) to 8.50 (CI 7.03–10.19). In those diagnosed in the 1960s significantly raised SMRs extended more than 15 years after diagnosis, consistent with the time course reported for late radiation effects (Zagars *et al*, 2004). These long-term risks do not appear to extend to those diagnosed after 1970. However, these findings should be interpreted with caution in view of the limited number of patients with long-term follow-up.

Fossa *et al* (2004) reported mortality rates in patients treated in Norway below age 56 years between 1962 and 1997, and found that among those not dying from malignant germ cell tumours there were significantly increased SMRs for diseases of the circulatory system, benign gastrointestinal disorders, and non germ cell malignancies. However, the mortality from non germ cell cancers was higher in those treated between 1970 and 1997 than in those treated in the 1960s.

Treatment patterns for seminomas have been somewhat distinct from those for nonseminomas. In the past most seminomas were managed with radiotherapy following orchidectomy whereas with nonseminomas, since the development of effective chemotherapy during the late 1970s, radiotherapy has been used increasingly rarely (Horwich *et al*, 2006). The radiation field most commonly used in seminoma patients included the para-aortic and ipsilateral pelvic lymph nodes. However, particularly during the early period of the cohort, selected patients also had radiotherapy to mediastinal and supraclavicular lymph nodes (Zagars and Babaian, 1987; Hanks *et al*, 1992). After seminomas we found an increased risk of both leukaemia (SIR 3.01; CI 1.44–5.54) and testis cancer (SIR 6.69; CI 4.28–9.95) in the first decade after diagnosis. In the second decade there were significantly increased risks for cancers of the pancreas (SIR 2.91; CI 1.26-5.73) and testis (SIR 3.96; CI 1.08-10.14). After more than 20 years there were significantly increased SIRs for pancreatic cancer (SIR 5.48; CI 2.37–10.80), colon cancer (2.36; CI 1.13–4.35), soft tissue sarcoma (SIR 13.64; CI 1.65–49.28), bladder cancer (SIR 4.28; CI 2.28–7.31), and leukaemia (SIR 4.48; CI 1.64–9.75). Considering all cancer sites combined, we found no overall excess risk in the first two decades, but more than 20 years from diagnosis the risk of developing a second cancer was doubled to 2.18 (95% CI 1.79–2.64).

Hay *et al* (1984), analysing second cancer risk in 547 patients treated with radiotherapy for testis cancer between 1950 and 1969 in Scotland, found an increased second cancer risk peaking 15–19 years after treatment. A long-term study from Norway of 365 seminoma patients treated with radiotherapy (Fossa *et al*, 1989) found an excess of lung cancer but not of leukaemia. A similar study from the MD Anderson Hospital (Zagars *et al*, 2004) found a cancer specific SMR of 1.9, which again was only significant at time periods more than 15 years after treatment. Second testicular cancers are likely to be because of factors other than treatment, and in fact the incidence is reduced after chemotherapy (Oliver *et al*, 2005), or after direct radiotherapy to the testis (Read, 1987; Dieckmann *et al*, 1993).

In patients treated for nonseminomas of the testis, we found an increased risk of developing leukaemia during the first decade (SIR 6.78; CI 2.93–13.36). The only significant excess risk observed during the second decade was for testis cancer (SIR 5.94; CI 2.18–12.93). After 20 years from diagnosis there were significant excesses of stomach (SIR 5.13; CI 1.40–13.13), rectum (SIR 4.49; CI 1.22–11.51) and pancreas cancers (SIR 10.17; CI 3.73–22.13). For all sites combined there was again a significant excess of second cancers only after more than 20 years from diagnosis (SIR 1.97; CI 1.40–2.69). In the past, these patients were often treated with both chemotherapy and radiotherapy (Peckham *et al*, 1979), making it difficult to relate risk to specific treatment modalities.

Our findings are largely consistent with those reported in long-term survivors of testicular cancer from 14 population-based cancer registries in North America and Europe (Travis *et al*, 2005). As here, the relative risk of second cancers 10 years or more after diagnosis was approximately doubled for both seminomas and nonseminomas. In addition to the sites for which we observed an excess, they found significant excesses for cancers of the pleura, lung and oesophagus. These workers also found lower risk of second cancers in patients with nonseminoma diagnosed after 1975. A nested case–control analysis from eight population-based cancer registries suggested that leukaemia risk after testicular cancer was related to both radiation dose to active bone marrow, and to cumulative dose of cisplatin (Travis *et al*, 2000). The relative risk was threefold after a total cumulative cisplatin dose of 650 mg, which would commonly be achieved with the standard four cycles of chemotherapy. An analysis of 1909 cases in The Netherlands diagnosed from 1971 to 1985 (van Leeuwen *et al*, 1993), based on more detailed treatment information, was able to link an increased risk of stomach cancer to radiation; this study found no excess of second malignancies after chemotherapy.

Our study has a number of shortcomings. It is based on cancer registry data, which contain limited treatment information largely confined to initial management and lacking detail of specific drugs, radiation doses, and radiation fields. Also, there may be under-reporting of second cancers in those who leave the registry catchment area. In the majority of such cases, the registry would not be informed of emigration, nor receive notification of subsequent cancers. This would lead to an underestimation of the risk of subsequent cancer, so that SIRs will be conservative. Additionally, as the data were extracted in 2005, incident cancers to the end of 2004 would still be a few percent incomplete, which again would tend to lead to an underestimation of SIRs, although here restricted to the short-term estimates.

In modern oncology practice, with better perception of individual prognosis, the overall burden of treatment for patients with testicular germ cell cancer has reduced considerably (Horwich *et al*, 2006). For example, many patients with stage I cancers are managed by surveillance after orchidectomy and thus mainly avoid both radiotherapy and chemotherapy (Warde *et al*, 2002; Horwich *et al*, 2006). This trend of reducing the toxicity of management should continue to reduce health risks in testicular cancer survivors.

## Figures and Tables

**Table 1:** SMRs by type of testis cancer, period of follow-up, and period of diagnosis

		**Time since diagnosis (years)**											
		**0–4**				**5–14**				**15+**			
				**95% CI**				**95% CI**				**95% CI**	
**Type**	**Period of diagnosis**	**Deaths**	**SMR**	**L**	**U**	**Deaths**	**SMR**	**L**	**U**	**Deaths**	**SMR**	**L**	**U**
*Seminomas*	1960–1969	94	8.62	6.96	10.54	51	1.89	1.41	2.48	91	2.55	2.05	3.13
	1970–1979	86	5.76	4.60	7.11	47	1.25	0.92	1.66	104	0.99	0.81	1.20
	1980–1989	69	2.59	2.02	3.28	79	0.99	0.78	1.23	51	0.93	0.69	1.22
	1990–2004	91	1.69	1.36	2.08	57	1.00	0.76	1.30	—	—	—	—
													
*Nonseminomas*	1960–1969	126	56.76	47.28	67.58	11	2.10	1.05	3.75	22	4.44	2.79	6.73
	1970–1979	241	45.13	39.61	51.20	25	1.66	1.07	2.44	52	1.17	0.87	1.53
	1980–1989	107	15.09	12.37	18.24	30	1.37	0.92	1.95	21	1.31	0.81	2.01
	1990–2004	117	8.50	7.03	10.19	18	1.26	0.75	2.00	—	—	—	—

SMRs=standardised mortality ratios; CI=confidence interval; L=lower; U=upper.

**Table 2:** SIRs for subsequent cancers by period of follow-up – seminomas

	**Time since diagnosis (years)**											
	**0–9**				**10–19**				**20+**			
			**95% CI**				**95% CI**				**95% CI**	
**Site**	**No.**	**SIR**	**L**	**U**	**No.**	**SIR**	**L**	**U**	**No.**	**SIR**	**L**	**U**
Stomach	8	1.60	0.69	3.15	1	0.24	0.01	1.32	4	1.86	0.51	4.76
Colon	7	0.88	0.35	1.81	6	0.83	0.30	1.81	10	2.36	1.13	4.35
Rectum	5	1.13	0.37	2.65	4	1.03	0.28	2.64	5	2.38	0.77	5.56
Pancreas	3	0.96	0.20	2.82	8	2.91	1.26	5.73	8	5.48	2.37	10.80
Lung	9	0.42	0.19	0.80	16	0.86	0.49	1.40	13	1.37	0.73	2.34
Soft tissue	1	1.81	0.05	10.11	2	5.82	0.70	21.01	2	13.64	1.65	49.28
Prostate	9	0.75	0.34	1.43	14	1.02	0.56	1.72	17	1.68	0.98	2.68
Testis	24	6.69	4.28	9.95	4	3.96	1.08	10.14	1	6.25	0.16	34.82
Kidney	3	1.08	0.22	3.14	2	0.84	0.10	3.04	2	1.65	0.20	5.97
Bladder	6	0.95	0.35	2.07	3	0.53	0.11	1.56	13	4.28	2.28	7.31
NHL	8	1.53	0.66	3.01	5	1.36	0.44	3.17	2	1.18	0.14	4.27
Leukaemia	10	3.01	1.44	5.54	1	0.39	0.01	2.18	6	4.48	1.64	9.75
Other^a^	16	0.54	0.31	0.88	15	0.65	0.36	1.07	23	2.01	1.28	3.02
Total^a^	109	1.04	0.85	1.25	81	0.91	0.72	1.13	106	2.18	1.79	2.64

NHL=Non-Hodgkin's lymphoma; SIRs=standardised incidence ratios.

a Excluding nonmelanoma skin cancer.

**Table 3:** SIRs for subsequent cancers by period of follow-up–non seminomas

	**Time since diagnosis (years)**											
	**0–9**				**10–19**				**20+**			
			**95% CI**				**95% CI**				**95% CI**	
**Site**	**No.**	**SIR**	**L**	**U**	**No.**	**SIR**	**L**	**U**	**No.**	**SIR**	**L**	**U**
Stomach	0	0.00	0.00	3.26	1	0.87	0.02	4.84	4	5.13	1.40	13.13
Colon	3	1.58	0.33	4.61	4	1.93	0.53	4.95	4	2.41	0.66	6.17
Rectum	1	0.94	0.02	5.26	2	1.72	0.21	6.23	4	4.49	1.22	11.51
Pancreas	0	0.00	0.00	5.05	2	2.50	0.30	9.03	6	10.17	3.73	22.13
Lung	4	0.89	0.24	2.28	3	0.62	0.13	1.80	5	1.45	0.47	3.38
Soft tissue	0	0.00	0.00	14.76	1	6.74	0.17	37.55	0	0.00	0.00	52.70
Prostate	2	0.95	0.11	3.42	2	0.66	0.08	2.38	3	0.80	0.16	2.33
Testis	4	1.52	0.41	3.88	6	5.94	2.18	12.93	0	0.00	0.00	20.49
Kidney	3	4.00	0.82	11.69	0	0.00	0.00	4.55	1	1.75	0.04	9.77
Bladder	3	2.11	0.44	6.17	2	1.33	0.16	4.82	1	0.93	0.02	5.16
NHL	2	1.03	0.12	3.72	1	0.64	0.02	3.55	2	2.30	0.28	8.30
Leukaemia	8	6.78	2.93	13.36	1	1.09	0.03	6.06	0	0.00	0.00	6.47
Other^a^	11	1.13	0.56	2.02	8	0.96	0.41	1.89	9	1.69	0.77	3.20
Total^a^	41	1.40	1.00	1.90	33	1.20	0.83	1.69	39	1.97	1.40	2.69

NHL=Non-Hodgkin's lymphoma.

a Excluding nonmelanoma skin cancer.
